# Health Tract, No. 169

**Published:** 1865

**Authors:** 


### MEDICAL ITEMS.

To produce sufficient light in internal cavities, to guide the surgeon in his operations, introduce a helix-formed glass tube of a very small bore, and burn by electricity any white light-producing compound, as carburetted hydrogen, carbonic acid, hydrochloric acid, etc.

BREAD.—Chemistry tells us that the best and most healthful bread is made by mixing flour, water, and yeast, by kneading it so effectually that the yeast and water shall come in contact with every grain of the flour, otherwise the bread will be bad; holes will be in it, and the crust will be easily detached from the soft part. Bad bread will be made out of the very best materials unless the kneading has been most thoroughly performed.

A CARROT-HEAD cut off a little below the top, and put in a basin of water, puts forth leaves, and makes a handsome ornament.

SMOKED HAM.—To give any ham the "smoky" taste, mix equal parts of vinegar and tar; dip the ham into it for a few minutes, then pour off and broil.

WORK.—In past times the world was worked too hard, and the masses did not live thirty years. Now human ingenuity has devised labor-saving machinery, so as to allow more time for rest, for recreation, and the cultivation of the social qualities of our nature; as witness the statement of that most ably conducted paper, the *Scientific American*, of New-York, to wit:

COTTON.—One man can spin more cotton-yarn now than four hundred men could have done in the same time in 1769, when Arkwright, the best cotton-spinner, took out his first patent.

FLOUR.—One man can make as much flour in a day now as a hundred and fifty could a century ago.

LACE.—One woman can make now as much lace in a day as a hundred women could a hundred years ago.

SUGAR.—It now requires only as many days to refine sugar as it did months thirty years ago.

LOOKING-GLASSES.—It once required six months to put quicksilver on a glass; now it needs only forty minutes.

ENGINES.—The engine of a first-rate iron-clad frigate will perform as much work in a day as forty-two thousand horses.

BUTTER may be kept sweet for many months thus: When first churned, wash it well in three waters; work it well again before packing; put it in large stone jars; dig a hole under any floor or in a cellar, leaving the top of the jar just above the ground; cover the butter two or three inches deep with strong brine, adding more butter until the jar is nearly full.

GREASY PEOPLE, fat and rubicund, are generally good-natured. Whether their greasiness is alike promotive of health and genial humor, is not here discussed. But grease is a "prophylactic," as doctors say; that is, it promotes health. It has passed into history that as often as the plague has decimated Smyrna, Constantinople, and other parts of the Levant, not a single case has ever been recorded of a person employed in loading and unloading oil being attacked even, let alone dying. The men know this so well that they freely offer to carry the sick of the plague to the hospitals. Wool-carders, who work in greased wool from morning until night (the trade of President Fillmore and the writer) are proverbially free from consumptive disease. Some of the African tribes expose themselves with impunity to the fervent heat of the desert when they have oiled themselves all over. Grease is great!

